# Reliability and Validity of the Korean version of the Center for Neurologic Study Bulbar Function Scale (K-CNS-BFS): An observational study

**DOI:** 10.1097/MD.0000000000038216

**Published:** 2024-06-21

**Authors:** Bu Kyung Park, Seong-il Oh, Minsung Kang, Hung Youl Seok, Jin-Mo Park, Sohyeon Kim, Hye-In Kim, Ji-Ah Kim, Jin-Sung Park

**Affiliations:** a College of Nursing, Kyungpook National University, Daegu, South Korea; b Department of Neurology, Kyung Hee University Hospital, Kyung Hee University College of Medicine, Seoul, South Korea; c Department of Neurology, Kyungpook National University Chilgok Hospital, Daegu, South Korea; d Department of Neurology, Dongsan Hospital, Keimyung, University School of Medicine, Daegu, South Korea; e Department of Neurology, Dongguk University College of Medicine, Dongguk University Gyeongju Hospital, Gyeongju, South Korea; f Department of Neurology, School of Medicine, Kyungpook National University, Kyungpook National University Chilgok Hospital, Daegu, South Korea; g Brain Science & Engineering Institute, Kyungpook National University, Daegu, South Korea.

**Keywords:** Amyotrophic lateral sclerosis, bulbar function, CNS-BFS, patient-reported outcome, reliability and validity

## Abstract

Bulbar dysfunction in amyotrophic lateral sclerosis (ALS) significantly affects daily life, leading to weight loss and reduced survival. Methods for evaluating bulbar dysfunction, including videofluoroscopic swallowing studies and the bulbar component of the ALS Functional Rating Scale-Revised (ALSFRS-R), have been employed; however, Korean-specific tools are lacking. The Center for Neurologic Study Bulbar Function Scale (CNS-BFS) comprehensively evaluates bulbar symptoms. This study aimed to develop and validate the Korean version of the CNS-BFS (K-CNS-BFS) to assess bulbar dysfunction in Korean patients with ALS. Twenty-seven patients with ALS were recruited from a tertiary hospital in South Korea based on revised El Escorial criteria. Demographic, clinical, and measurement data were collected. The K-CNS-BFS was evaluated for reliability and validity. Reliability assessment revealed strong internal consistency (Cronbach alpha) for the K-CNS-BFS subscales and total score. Test–retest reliability showed significant correlation. Content validity index was excellent, and convergent validity demonstrated significant correlations between the K-CNS-BFS and relevant measures. Discriminant validity was observed between the K-CNS-BFS and motor/respiratory subscores of the ALSFRS-R. Construct validity demonstrated significant correlations between the K-CNS-BFS subscales and total score. This is the first study to investigate the reliability and validity of the Korean version of the CNS-BFS, which showed consistent and reliable scores that correlated with tests for bulbar or general dysfunction. The K-CNS-BFS effectively measured bulbar dysfunction similar to the original CNS-BFS. The K-CNS-BFS is a reliable and valid tool for assessing bulbar dysfunction in patients with ALS in South Korea.

## 1. Introduction

Amyotrophic lateral sclerosis (ALS) is a neurodegenerative disease characterized by progressive motor weakness and atrophy due to motor neuron degeneration. Clinically, ALS can be divided into bulbar or limb onset based on the start of motor neuron degeneration in the brainstem of spinal motor neurons.^[1–4]^

Bulbar dysfunction is an important factor that influences activities of daily life, and can lead to tubal feeding or gastrostomy. Bulbar dysfunction causes significant weight loss and eventually reduces the survival and quality of life.^[2,5–9]^

Therefore, evaluation of bulbar dysfunction is clinically important for improving bulbar function, preventing long-term complications, and planning further treatment.^[6,9–11]^ In order to evaluate bulbar dysfunction, clinical, radiological, and electrophysiological assessments are required. Multidisciplinary approaches are immediately needed in addition to a neurological approach, and rehabilitation or procedures the evaluation must be considered.^[6,12,13]^

The video fluoroscopic swallowing study (VFSS) is frequently applied as an index for self-reported questionnaires to confirm bulbar dysfunction, but they may be associated with risks related to direct aspiration and asphyxia.^[6,13]^ The ALS Functional Rating Scale-Revised (ALSFRS-R) or Appel ALS Rating scale (AALSRS), which includes dysphagia and/or speech in the general assessment, is mainly used as a screening measure^[14]^; the Neuromuscular Disease Swallowing Status Scale and Oral Secretion Scale, which individually assess swallowing, saliva control, and speaking dysfunction, are also used.^[6,14]^ Among them, Center for Neurologic Study Bulbar Function Scale (CNS-BFS) has been validated as a screening method that can identify important elements of bulbar symptoms, including speech, swallowing, and salivation.^[12,15,16]^ In Korea, there is a demand for bulbar dysfunction-specific indicators, no study other than the general K-ALSFRS exists that has been validated in the Korean language for the evaluation of bulbar dysfunction in ALS patients.^[14]^

Therefore, in this study, we translated the CNS-BFS into a Korean version (K-CNS-BFS) and evaluated the reliability and validity of the K-CNS-BFS in Korean patients with ALS.

## 2. Methods

### 2.1. Participants

Patients diagnosed with ALS based on the revised El Escorial criteria were recruited from a tertiary university hospital specializing in motor neuron diseases in South Korea. Inclusion criteria were patients who were (1) aged 18 years or older, (2) clinically probable and clinically definite ALS, and (3) patients who had bulbar symptoms. This study was approved by the Institutional Review Board of Kyungpook National University Chilgok Hospital (KNUCH 2023-03-029).

The participants were recruited using convenience and purposive sampling in the outpatient department. When patients with ALS attended their clinic appointments, a research assistant thoroughly explained the study, and a written consent form was obtained from those who agreed to participate. The survey included demographic questions and the K-CNS-BFS. For test–retest reliability, the K-CNS-BFS was reevaluated within 2–4 weeks of the first administration.

### 2.2. Measurement

#### 2.2.1. Demographic, clinical data, VAS, and ALSFRS-R

Participants’ age, sex, disease duration from symptom onset (months), onset site (limb or bulbar), forced vital capacity (FVC), and ALSFRS-R scores^[14]^ were collected by reviewing medical records. In addition, the visual analog scale (VAS) scores for speech, swallowing, and salivation were surveyed. Penetration–aspiration scale (PAS) data were collected from the VFSS, which is the gold standard for dysphagia assessment.

#### 2.2.2. CNS-BFS

The CNS-BFS is composed of 21 items measuring bulbar function in ALS on a 5-point Likert scale (1 = does not apply, 5 = applies most of the time), with possible total scores ranging from 21 to 112, where a higher score indicates more impaired bulbar function.^[12]^ The CNS-BFS evaluates 3 domains of bulbar function: the 3 subscales measure speech, swallowing, and salivation. The subscales use self-report measures, and participants were asked to answer 7 items on each subscale. Regarding the speech subscale, participants who were unable to communicate by speaking were assigned a value of 6 for each item.

#### 2.2.3. Translation of the CNS-BFS into Korean

The CNS-BFS was translated following the process of translation and adaptation of the instrument proposed by the World Health Organization^[17]^: (1) forward translation; (2) expert panel for content validity index (CVI); (3) back translation; (4) pretesting for reliability and validity, which is the result of this study; and (5) the final version.

After obtaining permission from the original developers of the CNS-BFS (Dr Richard Smith from the Center for Neurologic Study), 2 authors fluent in English independently conducted the forward translation and compared the translated instrument. Next, an expert panel comprising 6 professors of neurology and ALS experts evaluated the translated instruments. All 6 members of the expert panel rated each item of the K-CNS-BFS in terms of its relevance to the underlying construct on a 4-point scale (1 = not relevant, 4 = highly relevant) for CVI calculations.^[18]^ Additionally, the expert panel commented on each item if they had any suggestions or questions. Through expert panel discussions, the translated CNS-BFS was revised and the K-CNS-BFS was produced. Subsequently, back translation was performed by 1 of the authors and compared with the original English CNS-BFS. Discrepancies were discussed with the authors and the final version of the K-CNS-BFS was produced (Appendix: K-CNS-BFS).

### 2.3. Statistical analysis

IBM SPSS version 28 (IBM Corp., Armonk, NY, USA) was used for statistical analyses. Descriptive statistics were used to explore the demographic and clinical characteristics of participants. If there was a missing value, the SPSS estimation method was used to replace it (linear trend at that point).

#### 2.3.1. Reliability

Internal consistency was calculated using Cronbach alpha for each item and if an item was deleted from the scale. The scale’s stability was evaluated by test–retest reliability testing using Pearson correlation coefficients.

#### 2.3.2. Validity

To determine the CVI for individual items, 6 members of the expert panel rated each item on a 4-point scale. Individual CVI was calculated as the number of experts who gave a rating of either 3 or 4, divided by 6 (the proportion of agreement about relevance). The CVI was calculated as the mean of individual CVIs for all items. Convergent validity was examined using Pearson correlation or Spearman rank-order correlation between the K-CNS-BFS and (1) VAS sialorrhea, speech, and swallowing; (2) ALSFRS-R-K bulbar subscale; (3) PAS; and (4) FVC. Discriminant validity was evaluated using Pearson correlation between the K-CNS-BFS and the ALSFRS-R-K motor and respiratory subscales. Construct validity was examined using domain-to-domain and domain-to-total correlations using Pearson correlation.

## 3. Results

### 3.1. Demographics

A total of 27 patients were recruited from the Department of Neurology in Seoul, Korea. The participants’ characteristics are presented in Table 1. Patients were 61 years old on average, primarily male (74.1%), 45.44 months of disease duration, and included 81% of limb-onset ALS patients. The mean PAS score and FVC % were 3.16 ± 1.75 and 67.78 ± 20.11, respectively. The mean K-CNS-BSF total score was 58.19 ± 27.96, and the mean ALSFRS-R-K total score was 25.63 ± 11.25.

**Table 1:** Clinical and demographic characteristics of participants (n = 27).

Characteristics [score range]	Mean ± SD/N (%)
Age, years	61.22 ± 9.55
Sex	
Male	20 (74.1)
Female	7 (25.9)
Disease duration, months	45.44 ± 39.77
Onset site	
Limb onset	22/27 (81%)
Bulbar onset	5/27 (19%)
PAS	3.16 ± 1.75
FVC (%)	67.78 ± 20.11
K-CNS-BFS Total score (21 items) [21–112]	58.19 ± 27.96
Sialorrhea (7 items) [7–35]	13.04 ± 7.01
Speech (7 items) [7–42]	23.85 ± 13.52
Swallowing (7 items) [7–35]	21.30 ± 9.60
VAS Sialorrhea [0–10]	3.74 ± 3.47
VAS Speech [0–10]	6.15 ± 3.65
VAS Swallowing [0–10]	5.22 ± 3.61
ALSFRS-R-K Total score (12 items) [0–48]	25.63 ± 11.25
Bulbar score (sum item 1–3) [0–12]	7.41 ± 3.54
Motor score (sum item 4–9) [0–24]	9.26 ± 6.48
Respiratory score (sum item 10–12) [0–12]	8.96 ± 3.79

ALSFRS-R = amyotrophic lateral sclerosis functional rating scale, FVC = forced vital capacity, K-CNS-BFS = Korean version of the Center for Neurologic Study Bulbar Function Scale, PAS = penetration–aspiration scale, VAS = visual analog scale.

### 3.2. Reliability

#### 3.2.1. Reliability: Internal consistency by Cronbach alpha

The internal consistency of the K-CNS-BFS was adequate, with Cronbach alpha values for the sialorrhea, speech, and swallowing subscales and the total scale being .862, .972, .901, and .964, respectively. The removal of any single item did not improve the overall Cronbach alpha of the K-CNS-BFS (Table 2).

**Table 2:** Internal consistency of the K-CNS-BFS.

Variable	Mean ± SD	Cronbach alpha if item deleted
Sialorrhea 1	2.67 ± 1.754	.962
Sialorrhea 2	1.00 ± .000	.966
Sialorrhea 3	1.56 ± 1.188	.964
Sialorrhea 4	1.33 ± 1.000	.964
Sialorrhea 5	1.89 ± 1.396	.964
Sialorrhea 6	2.00 ± 1.569	.961
Sialorrhea 7	2.59 ± 1.716	.961
Sialorrhea Subscale	Cronbach α = .862	
Speech 1	3.37 ± 2.115	.960
Speech 2	3.56 ± 2.082	.960
Speech 3	3.41 ± 2.117	.960
Speech 4	2.37 ± 2.115	.962
Speech 5	3.67 ± 2.094	.961
Speech 6	4.00 ± 1.961	.961
Speech 7	3.48 ± 2.119	.960
Speech Subscale	Cronbach α = .972	
Swallowing 1	3.00 ± 1.664	.961
Swallowing 2	3.74 ± 1.767	.962
Swallowing 3	2.96 ± 2.009	.961
Swallowing 4	2.22 ± 1.672	.961
Swallowing 5	4.22 ± 1.396	.963
Swallowing 6	2.74 ± 1.873	.963
Swallowing 7	2.41 ± 1.670	.962
Swallowing Subscale	Cronbach α = .901	
K-CNS-BFS Total	Cronbach α = .964	

K-CNS-BFS = Korean version of Center for Neurologic Study Bulbar Function Scale.

#### 3.2.2. Reliability: Test–retest reliability by Pearson correlation coefficients

Test–retest reliability was assessed with all 27 participants within 2–4 weeks of the pretest based on the patients’ next hospital appointment. Test–retest reliability was assessed with all 27 participants within 2–4 weeks of the pretest based on the patients’ next hospital appointment and a trained research assistant checked the participants’ outpatient appointment schedules ahead for the retest. Pearson correlation coefficients for test–retest reliability showed a strong and significant correlation (*r* = .996, *P* = .001). The acquired power with 27 sample size was 1.00.

### 3.3. Validity

#### 3.3.1. Content validity

The CVI was excellent (S-CVI = 0.91), scoring higher than 0.90.^[18]^ The overall individual CVI (I-CVI) was good, with scores higher than 0.78 except for 2 items (sialorrhea items 4 and 7). The I-CVI of sialorrhea subscale 7 (“My secretions are not manageable.”) was 0.67; therefore, the authors revised the sentence according to the experts’ suggestions. The I-CVI of sialorrhea subscale 4 (“Drooling causes me to be frustrated or embarrassed.”) was 0.16 at the first round of the CVI expert review. Therefore, the authors revised the item and reevaluated the I-CVI. The final I-CVI of item 4 improved to 0.67.

#### 3.3.2. Validity: Convergent validity by Pearson correlation coefficients between CNS-BFS, VAS, ALSFRS-R, and PAS

The convergent validity of the K-CNS-BFS was evaluated using the correlation among the VAS, ALSFRS-R bulbar subscale, PAS, and FVC (Table 3).

**Table 3:** Convergent validity and discriminant validity of the K-CNS-BFS.

	K-CNS-BFS			
	Sialorrhea	Speech	Swallowing	Total score
K-CNS-BFS				
Sialorrhea	1	.694†	.784†	.855†
Speech		1	.851†	.950†
Swallowing			1	.951†
VAS				
Sialorrhea	.900†	.658†	.692†	.782†
Speech	.691†	.844†	.754†	.840†
Swallowing	.702†	.671†	.849†	.792†
ALSFRS-R				
Bulbar subscale	−.837†	−.875†	−.936†	−.954†
Motor subscale	−.456*	−.272	−.361	−.392*
Respiratory subscale	−.477*	−.289	−.474*	−.499†
PAS‡	.340	.517†	.414*	.520†
FVC (%)‡	−.213	−.455*	−.292	−.376

ALSFRS-R = Amyotrophic Lateral Sclerosis Functional Rating Scale-Revised, FVC = forced vital capacity, K-CNS-BFS = Korean version of Center for Neurologic Study Bulbar Function Scale, PAS = penetration–aspiration scale, VAS = visual analog scale.

* *p* <.05.

† *p* <.01.

‡ Spearman rank-order correlation.

1. *Correlation between CNS-BFS and VAS:* The subscales of the K-CSN-BFS were significantly correlated with the corresponding VAS subscales: sialorrhea (*r* = .900, *P* < .01), speech (*r* = .844, *P* < .01), and swallowing (*r* = .849, *P* < .01). The acquired power with 27 sample size was 1.00 in all 3 subscales.
2. *Correlation between CNS-BFS total score and ALSFRS-R (bulbar subscale):* The total K-CNS-BFS score was highly correlated with the bulbar subscale of the ALSFRS-R (*r* = .954, *P* < .01). The K-CNS-BFS subscales for sialorrhea (*r* = −.837, *P* < .01), speech (*r* = −.875, *P* < .01), and swallowing (*r* = −.936, *P* < .01) subscales were also highly correlated with the bulbar subscale of ALSFRS-R-K.
3. *Correlation between CNS-BFS and PAS:* The K-CSN-BFS total score was significantly correlated with the PAS scores using Spearman rank-order correlation coefficients (rho = .520, *P* < .01).
4. *Correlation between CNS-BFS and FVC:* The K-CSN-BFS total score was not significant but showed a trend correlation with FVC percentages using Spearman rank-order correlation coefficients (rho = −.376, *P* = .053).

#### 3.3.3. Validity: Discriminant validity by Pearson correlation coefficients between K-CNS-BFS and ALSFRS-R (motor subscore, respiratory subscore)

The K-CNS-BFS total score showed a low correlation with the motor (r = -.392, *P* < .05) and respiratory subscores (*r* = −.499, *P* < .01) of the ALSFRS-R. Compared with the bulbar subscale of the ALSFRS-R, these motor and respiratory subscores showed a weaker relationship with the K-CNS-BFS total score. In addition, the motor and respiratory subscales did not significantly correlate with any of the subscales of the K-CNS-BFS (Table 3).

#### 3.3.4. Validity: Construct validity by domain-to-domain and domain-to-total correlations

The subscales of the K-CNS-BFS were significantly correlated with other domains as well as the total score (Table 3).

## 4. Discussion

In this study, we assessed the validity and reliability of the K-CNS-BFS by comparing it with the bulbar component of the Korean version of the ALSFRS-R and with PAS scores. The findings of this study show that the K-CNS-BFS measured the severity of bulbar dysfunction similarly to the original CNS-BFS.^[12]^ To the best of our knowledge, this is the first study to investigate the validity and reliability of the Korean version of CNS-BFS, especially for the assessment of bulbar dysfunction in ALS. The scores for the Korean version of the CNS-BFS were consistent and reliable, and correlated with the various subjective and objective tests for general or bulbar dysfunction utilized in the study; this suggests that it could be a useful measure of bulbar dysfunction in patients with ALS.

The ALSFRS-R is the most representative test method for evaluating the overall functional status of ALS and has been used to measure therapeutic efficacy in various clinical trials.^[10,19]^ In addition to the ALSFRS-R, the CNS-BFS is also used to effectively assess bulbar symptoms, but its use is less common compared to the ALSFRS-R and it is not considered an important clinical outcome measure.^[10]^ The ALSFRS-R evaluates 3 items (speech, swallowing, and salivation) reflecting bulbar function, and the total ALSFRS-R score is considered to have excellent reliability, but individual subscores (especially bulbar) do not have sufficient specificity for each organ.^[6,13]^ Therefore, the CNS-BFS was developed as a method to improve this limitation and focus on how bulbar function significantly impacts activities of daily living in patients with ALS.^[15]^ Meaningful results have been reported when evaluating bulbar dysfunction using the CNS-BFS compared to ALSFRS-R or VAS scores.^[12]^

The NEALS Bulbar Subcommittee recommends a practice protocol for evaluating bulbar dysfunction in patients with ALS. The Bulbar Subcommittee has suggested evaluating the CNS-BFS as a patient self-report questionnaire at the initial visit.^[13,20]^

The Cronbach alpha coefficient is a scale used to estimate reliability by determining the internal consistency within the test.^[21]^ A coefficient of 0.7 is considered to be acceptable reliability for the new scales. In this study, Cronbach alpha coefficients for the sialorrhea, speech, swallowing subscales, and the total scale of the K-CNS-BFS were .862, .972, .901, and .964, respectively. All coefficients were over .7 (Table 2), and therefore the internal consistency reliability of the K-CNS-BFS was satisfactory. Based on the results obtained when retesting within 2–4 weeks, the test–retest reliability of the total K-CNS-BFS score was also satisfactory. (*r* = .996, *P* = .001).

To confirm content validity, an expert panel of 6 neurologists specializing in ALS rated the CVI. The overall I-CVI was good for all but 2 items (sialorrhea items 4 and 7). The good scores were higher than the value of 0.78 suggested in a previous study.^[18]^ However, items 4 and 7 had low CVI scores; therefore, some items were modified and reevaluated. The CVI scores for the 2 items improved, but were lower than 0.78.

Convergent and discriminant validity were investigated by analyzing correlations between the K-CNS-BFS, VAS, total and bulbar subscales of the ALSFRS-R, and FVC. The K-CNS-BFS strongly correlated with the corresponding items on the VAS. The bulbar subscale of the ALSFRS-R score was strongly correlated with the subscales and total score of the K-CNS-BFS, but the motor subscale was less strongly correlated with the K-CNS-BFS.

The PAS, which indicates dysphagia function, correlated with speech, swallowing subscales, and total scores on the K-CNS-BFS; however, PAS did not correlate with sialorrhea. The FVC (%), which indicates respiratory function, was correlated with the speech subscale, but not with the other scores of the K-CNS-BFS. Assessment of bulbar function using PAS or self-reported outcomes with bulbar subscales of the ALSFRS-R were strongly correlated with the K-CNS-BFS, but assessments of respiratory function, such as the respiratory subscale of ALSFRS-R or FVC, were less correlated compared to bulbar dysfunction-related assessments. Discriminant validity was confirmed with a low correlation with items other than the bulbar function assessment. The subscales of the individual K-CNS-BFS were also significantly correlated, confirming construct validity.

This study had several limitations. First, although this study confirmed the bulbar function in ALS and validated the Korean version, the number of participants was small. However, considering the rarity of the disease, the scores correlated well with the previous bulbar scores. Second, although a wide range of patients with ALS based on the ALSFRS-R and disease duration were included, early or advanced patients were not evaluated; therefore, interpretation of the scores should be cautiously evaluated.

In conclusion, our results demonstrate that the K-CNS-BFS is a well-translated, reliable, and validated Korean version of the original CNS-BFS and hence could be a useful tool for the assessment of bulbar dysfunction in patients with ALS in South Korea.

## Author contributions

**Methodology:** Bu Kyung Park, Seong-il Oh, Jin-Sung Park.

**Project administration:** Bu Kyung Park, Seong-il Oh, Hye-In Kim.

**Software:** Bu Kyung Park.

**Validation:** Bu Kyung Park.

**Writing – original draft:** Bu Kyung Park, Seong-il Oh.

**Conceptualization:** Seong-il Oh, Jin-Sung Park.

**Formal analysis:** Seong-il Oh, Minsung Kang.

**Data curation:** Minsung Kang, Hung Youl Seok, Jin-Mo Park, Sohyeon Kim, Hye-In Kim, Ji-Ah Kim.

**Funding acquisition:** Jin-Sung Park.

**Investigation:** Jin-Sung Park.

**Supervision:** Jin-Sung Park.

**Writing – review & editing:** Jin-Sung Park.
